# Block Volume Estimation from the Discontinuity Spacing Measurements of Mesozoic Limestone Quarries, Karaburun Peninsula, Turkey

**DOI:** 10.1155/2014/363572

**Published:** 2014-02-13

**Authors:** Hakan Elci, Necdet Turk

**Affiliations:** ^1^Department of Geotechnics, Torbali Vocational School, Dokuz Eylul University, 35860 Izmir, Turkey; ^2^Department of Geological Engineering, Faculty of Engineering, Dokuz Eylul University, 35160 Izmir, Turkey

## Abstract

Block volumes are generally estimated by analyzing the discontinuity spacing measurements obtained either from the scan lines placed over the rock exposures or the borehole cores. Discontinuity spacing measurements made at the Mesozoic limestone quarries in Karaburun Peninsula were used to estimate the average block volumes that could be produced from them using the suggested methods in the literature. The Block Quality Designation (BQD) ratio method proposed by the authors has been found to have given in the same order of the rock block volume to the volumetric joint count (*J*_*v*_) method. Moreover, dimensions of the 2378 blocks produced between the years of 2009 and 2011 in the working quarries have been recorded. Assuming, that each block surfaces is a discontinuity, the mean block volume (*V*_*b*_), the mean volumetric joint count (*J*_*vb*_) and the mean block shape factor of the blocks are determined and compared with the estimated mean in situ block volumes (*V*_in_) and volumetric joint count (*J*_*vi*_) values estimated from the in situ discontinuity measurements. The established relations are presented as a chart to be used in practice for estimating the mean volume of blocks that can be obtained from a quarry site by analyzing the rock mass discontinuity spacing measurements.

## 1. Introduction 

Dimensions of the blocks that can be extracted from a rock mass are governed by the discontinuity planes present in it. Therefore, it is important to analyze the discontinuity spacing data to establish whether or not economical sized blocks can be obtained from a rock mass prior to commencing any quarrying operation. Analysis of the discontinuity spacing data does not only allow us to estimate the mean block volume but also enables us to determine whether the blocks can be economically extracted from a rock mass. Thus if the estimated mean block volume is small in a quarry site, in such case, it will not be economical to invest in such area.

There are several studies proposed in literature to estimate the mean block volume using the rock mass discontinuity spacing data. Priest and Hudson [1], Hudson [2] stated that the discontinuity planes show rather irregular patterns in terms of occurrence geometry and added that statistical methods need to be utilized for explaining these geometries and composing relevant models. Attewel and Farmer [3], Priest and Hudson [1], Hudson and Priest [4], Einstein et al. [5], and Ulusay and ve Sönmez [6] defined the geometry of the discontinuity planes in two-dimensional irregularly scattered lines and remarked that a negative exponential relation exists between the spacing and the frequency of the discontinuity planes which is expressed as follows:

(1) $$\begin{array}{c} f\left(S_{m}\right)=\lambda \cdot e^{-\lambda S_{m}}, \end{array}$$

where *f*(*S*_*m*_): probability; *λ*: mean discontinuity frequency; *S*_*m*_: mean discontinuity spacing.

Although the majority of the scientists who statistically analyzed the rock mass discontinuity spacing suggested a negative exponential distribution, Wang [7], Wittke [8], Barton and Zoback [9], Priest [10], Lu and Latham [11], and Doyuran et al. [12] suggested log normal distribution. On the other hand, Rouleau and Gale [13] suggested the use of Weibull distribution [14]. Hudson [2] noted that log normal and Weibull distribution results were consistent with each other and added that the results of obtained mean discontinuity spacing as a consequence of these distributions were considerably close to those mean discontinuity spacing values computed by the negative exponential equation, suggested by Hudson and Priest [4] and Priest and Hudson [1].

Similar statistical studies performed using discontinuity spacing measurement made in Turkey various rock masses, for block stone quarrying and rock mass classification. Engineering classification of rock masses, negative exponential distribution were found to apply to analysis of the discontinuity data from researchers like Ayday [15], Ulusay [16], Gökceoğlu [17], Yavuz et al. [18], Ulusay and ve Sönmez [6], Nefeslioglu et al. [19], and Kaya et al. [20].

In the literature, there are two common methods generally used for the estimation of block dimensions using the discontinuity spacing data. The first method is called the volumetric joint count method [21] and the latter is called the weighted joint density method [22].

The volumetric joint count (*J*_*v*_) is described as the number of joints encountered in a cubic meter of fractured rock mass and is the measurement of number of intersecting discontinuities within the fractured rock mass per 1 m^3^. *J*_*v*_ is usually calculated from the mean spacing value of joint sets;

(2) $$\begin{array}{c} J_{v}=\frac{1}{S_{1}}+\frac{1}{S_{2}}+\frac{1}{S_{3}}+\cdots +\frac{1}{S_{n}}, \end{array}$$

where *S*_*i*_ is the true mean discontinuity spacing for each discontinuity set, for *i* = 1, 2,…, *n*. *n* is the total number of discontinuities.

By using the discontinuity spacing values measured in one dimension and also the volumetric joint count (*J*_*v*_), Palmström [21] suggested the following relation between the in situ mean block volume (*V*_*i*_) and the volumetric joint cont (*J*_*v*_) to estimate in situ mean block volume (*V*_*i*_), from one-dimensional discontinuity spacing measurement:

(3) $$\begin{array}{c} V_{b}=\frac{\beta }{{\left(J_{v}\right)}^{3}}. \end{array}$$

*β* is defined as shape factor in (3) and was suggested to be equal *β* = 36 for the prismatic shape blocks Palmström [21].

Latham et al. [23] claimed that the angle between the drilling direction and discontinuity plane happens to be mostly 35° and suggested the calculation of in situ block volume (*V*_*i*_) from the mean joint spacing (*S*_*m*_) for general use by also assuming that the shape factor *β* = 36:

(4) $$\begin{array}{c} V_{i}=36{\left(\frac{S_{m}}{2}\right)}^{3}. \end{array}$$

Palmström [24] stated that the block volume in rock masses bearing three or more joint sets could also be estimated from the equation below using the discontinuity spacing and the angles between the joint sets

(5) $$\begin{array}{c} V_{i}=\frac{\left(S_{1}\times S_{2}\times S_{3}\right)}{\left(\mathrm{Sin}\gamma_{1}\times \mathrm{Sin}\gamma_{2}\times \mathrm{Sin}\gamma_{3}\right)}. \end{array}$$

If to a rock mass contains only two joint sets; (5) suggested to be used in the form of;

(6) $$\begin{array}{c} V_{i}\;=S_{1}\times S_{2}\times 5S_{1}. \end{array}$$

In (5) and (6), *V*_*i*_: estimated field block volume (m^³^); *S*_1_, *S*_2_, *S*_3_: spacing between the joint sets (m); *γ*_1_, *γ*_2_, *γ*_3_: inclination between the joint sets (°).

Rock mass block quality designation ratio concept has been proposed to assess the rock mass quality for block production quality based on the field discontinuity measurements [25]. Rock mass block quality designation (BQD) has been proposed as the ratio of the cumulative length of the discontinuity spacing and/or borehole core length equal to or greater than 1 m to the total length of the scan (trace) line or the borehole length as percentage:

(7) Rock  Mass  Block  Quality  Designation, BQD%=(ΣS≥1 mL)·100,

where *S* is the discontinuity spacing equal to or greater than 1.0 m and *L* is the measurement line length (m).

**Figure 1:**
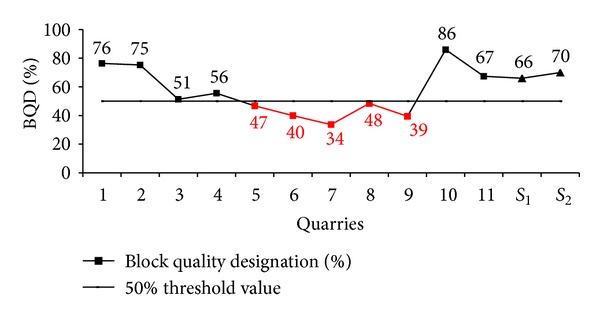
Rock mass block quality designation (BQD) ratios of the Karaburun limestone quarries. *S*_1_ and *S*_2_: working Süpren marble quarries (Nefeslioglu et al. [19]).

When the measured discontinuity spacings data of the limestone quarries of Karaburun peninsula are evaluated, it is observed that BQD ratio of 50% was found to the threshold value between working and abandoned the quarries. While the working quarries are noted to have the BQD ratio more than 50%, the abandoned quarries are noted to have BQD value less than 50% (Figure 1).

**Figure 2:**
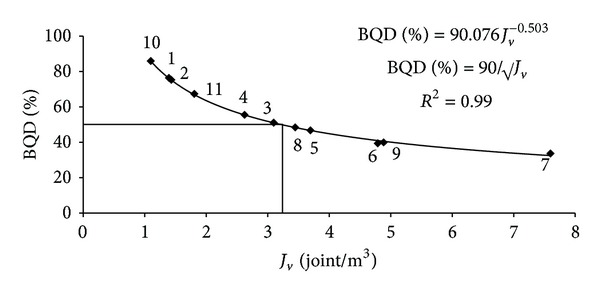
Relation between the rock mass block quality designation (BQD) and the volumetric joint count (*J*_*v*_) for the Karaburun limestone quarries.

A decreasing exponential relation has been obtained between the BQD ratios and *J*_*v*_ values of the working and abandoned quarries as shown in Figure 2. This relation has been found to be best represented by

(8) $$\begin{array}{c} \text{BQD}\%=\frac{90}{\surd J_{vi}},\;\left(R^{2}=0.99\right). \end{array}$$

Using (8) with (3) a relation between the mean in-situ block volume and the BQD ratio is obtained as

(9) $$\begin{array}{c} V_{i}=1.88\beta {\left(\text{BQD}\right)}^{6}\times 10^{-12} \end{array}$$

for the Karaburun limestone quarries. Taking *β* = 36; thus (8) becomes

(10) $$\begin{array}{c} V_{i}=6.77\times {\left(\text{BQD}\right)}^{6}\times 10^{-11}. \end{array}$$

In this study, initially the discontinuity spacings of the limestone quarry benches were determined by the photos of the quarry benches and their details were checked later in the field and then they were statistically analyzed (Figure 4). The mean true discontinuity spacings, average discontinuity spacing, and angles between the joint sets, were determined for each studied quarry and given in Tables 1 and 2. The volumetric joint count, and BQD values were determined for each quarry and their estimated values are given in Tables 1 and 2. By using these discontinuity spacing values, (Tables 1 and 2), mean in-situ block volumes of the each studied quarry using (1)–(10). Additionally, mean in situ block volume values (*V*_*i*_) obtained using various methods are also given in Table 3 for comparison with one another.

Moreover, the dimensions of 2378 limestone blocks produced between the years of 2009 and 2011 in the Karaburun limestone quarries were recorded and their volumes are calculated from their dimension measurements. Assuming that each block surface is a discontinuity, the volumetric joint count of the produced blocks (*J*_*v*_) are estimated. Since knowing the block volume (*V*_*b*_) and the volumetric joint count (*J*_*v*_). The shape factors (*β*_*b*_) of the limestone blocks were also calculated using (3). The *J*_*vi*_, BQD, and *V*_*i*_ values determined for quarries were compared with the *V*_*b*_, *J*_*vb*_, and *β*_*b*_ of the blocks produced from them. Additionally a graph was proposed to predict the mean trimmed block size, by analyzing the discontinuity spacing data measured in the field.

## 2. Karaburun Limestone Quarries

**Figure 3:**
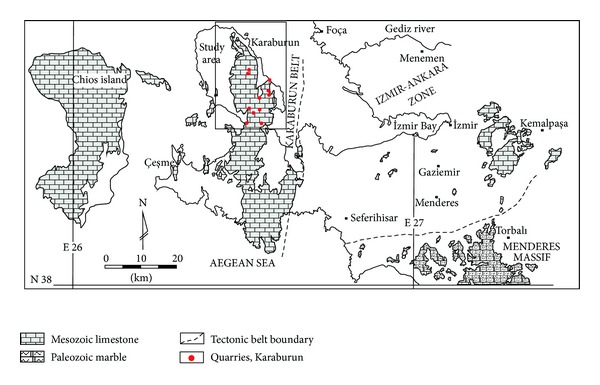
Location map of the study area and the outcrop of Mesozoic carbonate rocks in İzmir Province modified from Yakut [26] and Güngör and Erdoğan [27].

The Karaburun limestone quarries are located in the western part of Turkey with limited transportation access to the major cities (Figure 3). However, in recent years the increasing demands for dimension stone and aggregate have made people to invest in this part of Turkey. The quarries are opened up mainly in Triassic-Cretaceous aged limestone. The discontinuity maps of Karaburun limestone quarries have been prepared from the photographs taken at 1.5 m height keeping the camera vertical to the quarry benches and joining the photographs using the computer software “photostitch” and thus producing single photo for each quarry at A0 scale and the identified discontinuity traces were later checked in the field.

**Figure 4:**
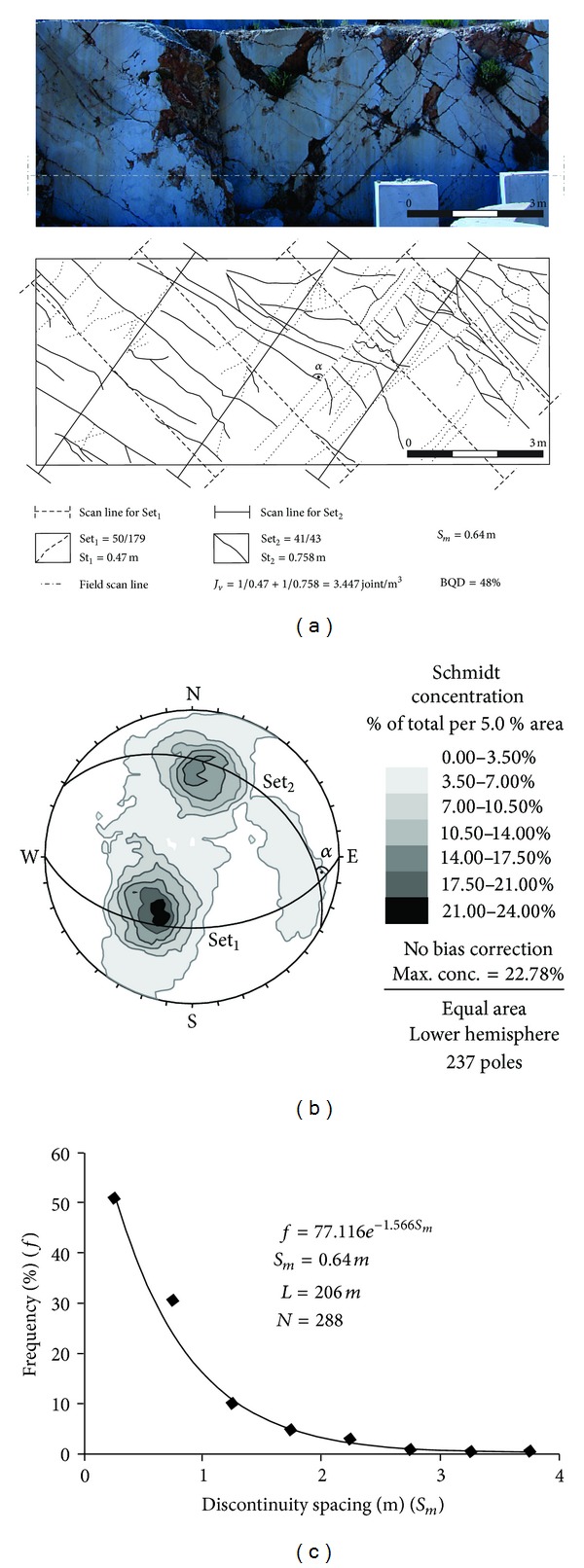
(a) A panoramic view and a section of 1/100 scale discontinuity trace map of a part of the quarry 8. (b) Lower hemispherical stereographic projection plots of the discontinuity traces in quarry 8. (c) Negative exponential distribution is found to fit into the discontinuity spacing data obtained along the horizontal trace line direction.

Discontinuity spacings are recorded in six active and five abandoned limestone quarries. The discontinuity spacing determinations have been made along 2199 m long line over the photos of 189 quarry benches (Figure 4). General properties of the discontinuities of the Karaburun Peninsula limestone quarries are given in Table 1. Additionally the orientations of discontinuities have been evaluated by plotting the dips and dip directions of discontinuity planes on the lower hemispherical projection (LHP) and the numbers and orientation of discontinuity sets were determined from the LHP analysis of the discontinuities for each limestone quarry. Discontinuity traces recorded at Quarry 8 and their analysis are given in Figure 4 as an example.

There are three discontinuity sets recorded in quarries 1, 2, 3, 4, 9, and 10 and two discontinuity sets recorded in quarries 5, 6, and 8. Quarries 7 and 11 have four discontinuity sets (Table 1).

The frequencies of the discontinuity spacings less than 1 and greater than 1 m are presented for the Karaburun limestone quarries in Table 2. The BQD ratio of the studied limestone quarries is also given in Table 1.

In situ mean block volumes estimated using the various suggested relations in literature are given in Table 3. The in situ block volume estimation method proposed by Elçi and Türk [25] are found to have given similar block volume values calculated using to the method suggested by Palmström [22] for the Karaburun limestone quarries (Table 3).

## 3. Dimension Analysis of the Extracted Blocks

In addition to the discontinuity measurements made at Karaburun the limestone quarries dimensions of 2378 limestone blocks which were produced between the years of 2009 and 2011 in the working quarries were also recorded. The mean volume of the blocks for each quarry calculated using the length, height, and width of the produced blocks is given in Table 4.

### 3.1. Volumetric Joint Count and Shape Factor of Blocks

By assuming that the surface of a limestone block is a discontinuity plane, the volumetric joint count (*J*_*vb*_) of a block can be calculated from (8) as given below:

(11) $$\begin{array}{c} J_{vb}=\frac{1}{\text{length}\left(l\right)}+\frac{1}{\text{width}\left(w\right)}+\frac{1}{\text{height}\left(h\right)}, \end{array}$$

Where *J*_*vb*_ = volumetric joint count of a limestone block (1/m^3^), length = length of limestone block (m), width = width of limestone block (m), and height = height of limestone block (m).

The volumetric joint count values of blocks (*J*_*vb*_) calculated from the dimensions of blocks produced at six working Karaburun quarries are given in Table 5. The weighted average *J*_*vb*_ value of 2378 blocks is found to be *J*_*b*_ = 1.868 joints/m^3^ and the weighted average block volume is calculated to be *V*_*b*_ = 4.127* *m^3^. By using these values in (3), the weighted average shape factor of the blocks produced in the Karaburun limestone quarries was found to be *β* = 29.17 (Table 5).

**Figure 5:**
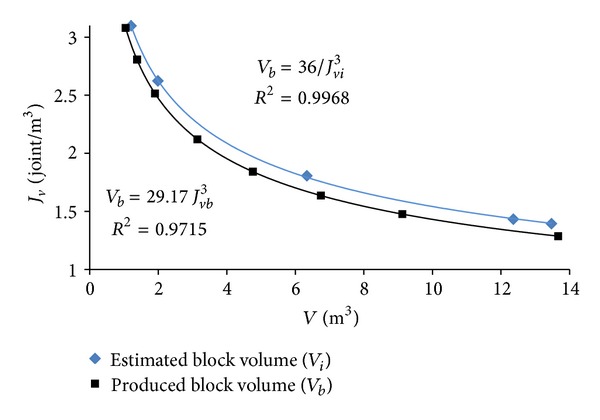
The relation between the volumetric joint counts, mean discontinuity spacings (*J*_*vi*_, *J*_*vb*_), and block volumes (*V*_*i*_, *V*_*b*_) of the Karaburun limestone.

Plot of the mean limestone block volume *V*_*b*_ versus the volumetric joint counts (*J*_*v*_) of the blocks extracted from Karaburun limestone quarries is given in Figure 5. *V*_*b*_ = 29.17/(*J*_*v*_)^3^ relation is obtained and plotted. In a similar manner, plot of the *V*_*i*_ = 36/(*J*_*v*_)^3^ relation is given for comparison in Figure 5. As seen in Figure 5, while the *J*_*v*_ value of the limestone blocks varies between 1 and 3, the block volume *V*_*b*_ values range from 1 to 14 m^3^.

## 4. Discussions

Before deciding to open a quarry in a from rock mass for block stone extraction, it is important to estimate the block volume as well as determining the engineering properties of the rock material. Since the block quarrying requires large amount capital, if the extractable block volume is predicted before starting quarrying operation, investments in an uneconomical sites can be prevented. In the Karaburun Peninsula, there are 5 quarries closed down soon after starting the quarrying operation. This situation is believed to be due to not carrying out sufficient discontinuities analysis of the rock mass to see whether or not economical blocks can be extracted from them before starting any quarrying operation in it.

In this study, the discontinuity spacing data obtained at 6 currently operating and 5 abandoned limestone quarries located in the Karaburun Peninsula (İzmir) and also the block dimensions obtained from the working quarries were statistically analyzed. The in-situ block volumes estimated using the mean discontinuity spacing, true discontinuity spacing, and volumetric joint count are found to be considerably different volume values from the produced block volumes (Table 2). The in situ block volumes calculated by the BQD, have given similar results to the Palmström's [22] method. Since the BQD method depends on the ratio of the cumulative discontinuity spacings greater than 1 m over the trace or scan line, it is more practical to use.

Munoz de la Nava [28] emphasized that no idea would be gained about the dimension of blocks confined by natural discontinuities with the help of *J*_*v*_ and added that *J*_*v*_ should be less than 3 so as to get proper blocks. Garcia [29] stated that *J*_*v*_ values should not exceed 2 in order to get convenient blocks from a rock mass. On the other hand, Sousa [30, 31] used this threshold value in the block stone quarrying of granitic rocks. The maximum value of *J*_*vi*_ was determined to be 3.10 joints/m^3^ in the working quarries in Karaburun Peninsula (Table 1). Additionally, the weighted mean *J*_*vb*_, of the blocks extracted from Karaburun limestone quarries, was found to be 1.926.

**Figure 6:**
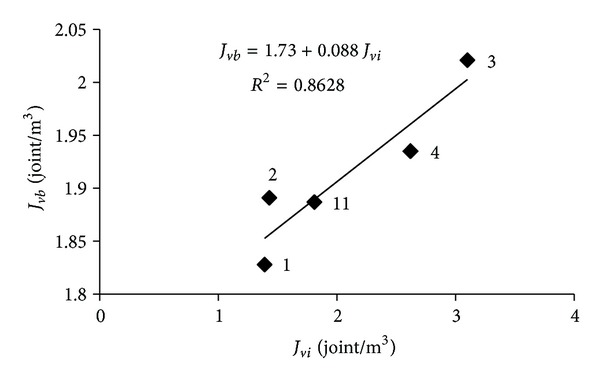
Mean in situ volumetric joint count (*J*_*vi*_) versus mean block volumetric joint count (*J*_*vb*_) for the Karaburun limestone.

*J*_*vi*_ values obtained from the discontinuity spacings in Karaburun limestones quarries and *J*_*vb*_ values obtained from the blocks dimensions are presented in Table 2. The correlation between in-situ *J*_*vi*_ values and block *J*_*vb*_ is also shown in Figure 6. There is a linear relation found between *J*_*vi*_ and *J*_*vb*_ as *J*_*vb*_ = 1.73 + 0.088*J*_*vi*_  (*R* ^2^ = 0.8628). This relation shows that the in situ field estimated *J*_*vi*_ value is lower than the block *J*_*v*_ value as expected.

**Figure 7:**
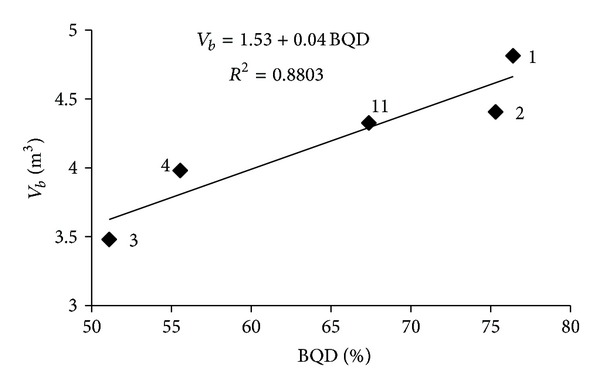
Relationship between the % BQD ratio and the mean volume of the blocks (*V*_*b*_) produced in the Karaburun limestone quarries.

**Figure 8:**
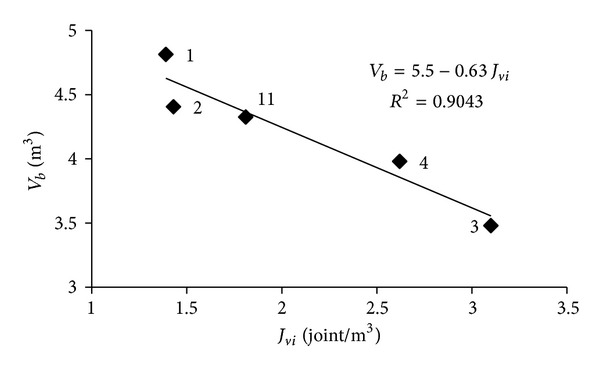
Relationship between the mean in-situ volumetric joint count (*J*_*vi*_) and the mean volumes of the blocks (*V*_*b*_) produced in the Karaburun limestone quarries.

A meaningful (*R* ^2^ = 0.8803) relation was obtained between *V*_*b*_ and BQD values in the form of *V*_*b*_ = 1.53 + 0.04BQD  (*R* ^2^ = 0.8803) (Figure 7). Additionally, *V*_*b*_ = 5.5 − 0.63*J*_*vi*_  (*R* ^2^ = 0.90) relation was established between the in situ *J*_*vi*_ and *V*_*b*_ block values as shown in Figure 8.

The sizes and/or weights of the blocks produced at quarries are constricted by the capacity of machinery and equipment on the one hand and by roadway transport on the other hand. Therefore, the average block volumes are trimmed to about 4-5 m^3^ in the quarries. Quarry 10 seems to be the best quarry in the Karaburun Peninsula both in terms of BQD and *J*_*v*_ values. However, as the capacity of the machines employed in this quarry had small capacity, the obtained maximum block volume extracted to be only 3.1 m^3^. For this reason, the discontinuity data of the quarry 10 was not used in the graphs presented in Figures 6 through 9.

**Figure 9:**
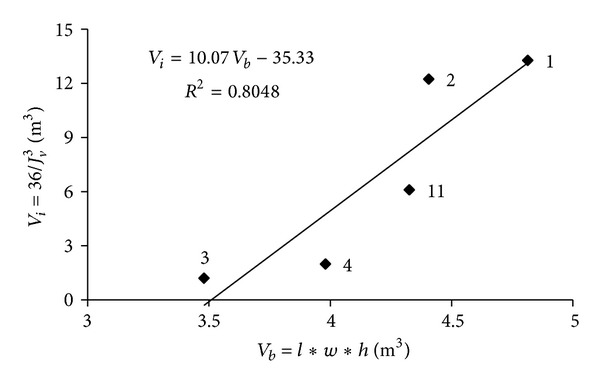
Relationship between the estimated in situ block volume (*V*_*i*_) and the mean volumes of the blocks (*V*_*b*_) produced in the Karaburun limestone quarries.

The relationships obtained between the discontinuity spacing data recorded in the field and the volume of the produced limestone blocks shown in Figures 6, 7, 8, and 9 will serve as a guide to the prospective quarry operators in determination of the block volumes that can would be obtained from a rock mass.

**Figure 10:**
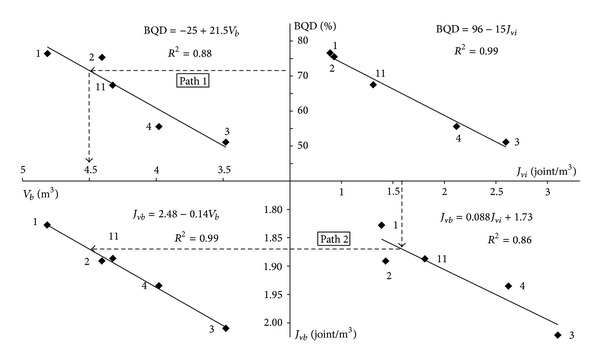
A general chart to estimate the mean block volume from the field discontinuity spacing data at the limestone quarries in Karaburun Peninsula.

A chart is presented in Figure 10 by using the various relations determined between the in situ discontinuity spacing data obtained at limestone quarries of the Karaburun Peninsula and the dimensions of limestone blocks produced in them (Figures 6, 7, 8, and 9). This graph can be used in two ways; either by determining the BQD of the rock mass first and then estimating the mean rock block volume from the established relation between the BQD ratio and block volume (Path 1) or by estimating the *J*_*vb*_ from the *J*_*vi*_ − *J*_*vb*_ relation and then determine rock block volume from the *J*_*vb*_ − *V*_*b*_ relation (Path 2) (Figure 10).

## 5. Conclusions

The following conclusions are reached with reference to the analysis of the discontinuities spacing measurement made at the limestone quarries in the Karaburun Peninsula and also the analyzing of the blocks dimensions produced in these quarries.

 Block Quality Designation method has been proposed for characterizing the rock mass quality for block production. BQD is a simple and an easy approach to use. The BQD method has found to give similar block volume values, as to the method suggested by Palmström (1995).
 The rock mass having BQD value > 50% is found to give economical blocks. On the other hand, the rock mass having BQD value less than 50% are found to be not suitable for economical block production.
 The *V*_*b*_, *J*_*vb*_ and *β* values obtained for the produced limestone blocks in the Karaburun limestone quarries by the assumption of each block surface being a discontinuity, have given smaller block volumes than the estimated block volumes using the field *J*_*vi*_ values.
 The proposed block shape factor *β* = 36 value for field block volume estimation by Palmström [21] was found to be *β* = 29.17 for the regular shaped blocks produced at the Karaburun limestone quarries.
 The relations established among the *BQD*, *J*_*vi*_, *J*_*vb*_, and *V*_*b*_ are presented as a graph for use in practice to estimate the regular shaped mean block volume obtainable from the in situ discontinuity measurements in a quarry site.

## Figures and Tables

**Table 1:** General properties of the discontinuities of Karaburun limestone quarries.

Quarry no.	Main discontinuity orientation		*γ* (°)	*S*_*t*_ (m)	*S*_*m*_ (m)	*J*_*v*_ (joint/m^3^)	BQD (% )
1	Set1	45/277	0.78	1.910	1.25	1.395	76
	Set2	66/187	0.64	2.013			
	Set3	71/344	0.70	2.668			

2	Set1	85/179	0.19	2.005	1.07	1.434	75
	Set2	84/0	0.98	1.975			
	Set3	18/320	0.93	2.330			

3	Set1	70/260	0.99	0.813	0.57	3.098	51
	Set2	22/80	0.93	1.002			
	Set3	70/357	0.94	1.151			

4	Set1	82/46	0.17	1.141	0.78	2.624	56
	Set2	88/90	0.95	1.184			
	Set3	21/46	0.94	1.108			

5*	Set1	33/26	0.64	0.504	0.68	3.696	47
	Set2	70/217		0.584			

6*	Set1	68/202	0.62	0.322	0.50	4.885	40
	Set2	75/29		0.562			

7*	Set1	65/90	0.74	1.010	0.50	7.598	34
	Set2	67/20	0.62	0.612			
	Set3	66/194	0.47	0.480			
	Set4	34/341	0.94	0.346			

8*	Set1	50/179	0.71	0.470	0.64	3.447	48
	Set2	38/29		0.758			

9*	Set1	22/173	0.99	0.485	0.56	4.791	39
	Set2	57/31	0.89	0.682			
	Set3	81/228	0.68	0.792			

10	Set1	30/36	0.99	2.160	1.60	1.099	86
	Set2	67/239	0.83	2.325			
	Set3	57/107	0.79	4.850			

11	Set1	80/74	0.64	3.470	1.14	1.807	67
	Set2	60/238	0.92	1.598			
	Set3	62/160	0.89	1.960			
	Set4	58/318	0.59	2.615			

*S*_*t*_: true discontinuity spacing; *S*_*m*_: mean discontinuity spacing; *J*_*v*_: volumetric joint count; BQD: block quality designation; *: abandoned quarries.

**Table 2:** Discontinuity spacing frequency of the Karaburun limestone quarries.

Quarry no.	1	2	3	4	5	6	7	8	9	10	11
	Discontinuity spacing frequency (%)										
<1 m.	59	57	82	77	77	84	86	78	81	39	63
≥1 m.	41	43	18	23	23	16	14	22	19	61	37

*L* (m)	247	117	440	118	83	131	119	206	136	162	440
Σ*S*_≥1_ (m)	190	88	225	67	39	52	40	100	54	139	296
BQD (%) = Σ*S*_≥1 m_/*L*	76	75	51	56	47	40	34	48	40	86	67

**Table 3:** Estimated in situ block volume of the Karaburun limestone quarries using the suggested methods in the literature.

Quarry no.	Palmström, 1995 (3)	Latham et al., 2006 (4)	Palmsröm, 2005 (5) and (6)	Elçi and Türk, 2013 (10)	Average produced block volume (*V*_*i*_, m^3^)
1	13.29	8.852	7.04	13.47	4.814 ± 2.187
2	12.23	5.482	16.57	12.36	4.406 ± 1.829
3	1.21	0.820	1.20	1.21	3.480 ± 1.381
4	1.99	2.135	2.9	1.99	3.980 ± 1.675
5	0.71	1.412	0.74	0.71	—
6	0.39	0.556	0.29	0.28	—
7	0.08	0.546	0.16	0.10	—
8	0.78	1.174	0.84	0.87	—
9	0.33	0.807	0.31	0.25	—
10	27.12	18.432	28.15	27.35	3.148 ± 1.645
11	6.10	6.221	10.14	6.33	4.026 ± 0.572

**Table 4:** Mean length, height, width, and volume of the limestone blocks produced at limestone quarries in the Karaburun limestone quarries.

Quarry no.	Mean block dimension, (m)			Block volume *V* = *l*·*w*·*h* (m^3^)	Number of blocks
	Length (*l*) (m)	Width (*w*) (m)	Height (*h*) (m)		
1	2.328 ± 0.496	1.298 ± 0.313	1.593 ± 0.265	4.814 ± 2.187	798
2	2.304 ± 0.516	1.225 ± 0.254	1.561 ± 0.276	4.406 ± 1.829	192
3	1.986 ± 0.400	1.205 ± 0.220	1.454 ± 0.223	3.480 ± 1.381	353
4	2.090 ± 0.332	1.248 ± 0.224	1.526 ± 0.264	3.980 ± 1.675	24
10	1.951 ± 0.400	1.151 ± 0.231	1.402 ± 0.213	3.148 ± 1.645	158
11	2.192 ± 0.426	1.274 ± 0.243	1.549 ± 0.191	4.026 ± 0.572	853

Weighted averaged	2.142 ± 0.145	1.234 ± 0.048	1.514 ± 0.066	4.127 ± 0.642	2378*

*: total number of blocks.

**Table 5:** The mean volumetric joint counts (*J*_*v*_) mean block volume and the shape factor (*β*) values for the limestone blocks produced in the Karaburun limestone quarries.

Quarry no.	Mean *J*_*v*_ (1/m^3^)	Mean *V*_*b*_ (m^3^)	Mean *β* = *V*·(*J*_*v*_)^3^ (m^3^)	Number of blocks
1	1.828 ± 0.301	4.814 ± 2.187	29.41	798
2	1.891 ± 0.310	4.406 ± 1.829	29.79	192
3	2.021 ± 0.309	3.480 ± 1.381	28.73	353
4	1.935 ± 0.264	3.980 ± 1.675	28.84	24
10	2.095 ± 0.461	3.148 ± 1.645	28.93	158
11	1.887 ± 0.368	4.026 ± 0.572	29.07	853
Weighted average	1.868 ± 0.100	4.127 ± 0.642	29.17	2378*

*: total number of blocks.
